# Specialist supportive clinical management (SSCM) for anorexia nervosa: Content analysis, change over course of therapy, and relation to outcome

**DOI:** 10.1186/2050-2974-3-S1-O1

**Published:** 2015-11-23

**Authors:** Virginia McIntosh

**Affiliations:** 1 Clinical Research Unit; University of Otago, New Zealand; 2 Christchurch & Canterbury District Health Board, New Zealand

## 

Specialist Supportive Clinical Management (SSCM) is a psychotherapy with promising potential for the treatment of anorexia nervosa. SSCM has two distinct components: clinical management, which involves alleviation of the symptoms of anorexia nervosa, particularly focusing on weight gain via resumption of eating; and a supportive psychotherapeutic approach to issues identified by the patient as important. The current study aimed to categorise, quantify and describe SSCM content across outcomes and over the course of therapy. SSCM therapy transcripts from ten participants in the original clinical trial of SSCM for anorexia nervosa were examined using thematic and content qualitative analyses. Clinical management content declined over time, whereas supportive psychotherapy content increased. Overall, there was more clinical management content for participants with a good outcome relative to participants with a poor outcome

